# Low dose Mifepristone (100 mg) for medical termination of pregnancy

**DOI:** 10.4102/phcfm.v3i1.254

**Published:** 2011-09-08

**Authors:** Shikha Seth, Arun Nagrath, Neeru Goel

**Affiliations:** 1 Department of Obstetrics & Gynaecology, U.P. Rural Institute of Medical Sciences & Research Saifai, Etawah, India; 2 Department of Obstetrics & Gynaecology, Era's Medical College, Sarfarazganj, Lucknow, India

## Abstract

**Background:**

Abortion is the most common entity in the practice of obstetrics and gynaecology. Different methods and modes have been opted for until now to find an effective regimen with the least complications. We have tried the minimal dose (100 mg) of Mifepristone (PO) instead of the presently recommended 200 mg for medical abortion in early first trimester cases.

**Objectives:**

The objective of the study was to determine the efficacy of low dose (100 mg) Mifepristone for medical termination of early pregnancy with oral Misoprostol 800 μg, 24 hours later.

**Design:**

A prospective analytical study was conducted on a population of 82 early-pregnant patients who have requested medical abortions.

**Method:**

Pregnant women of less than 56 days gestation age from their last menstrual period, requesting medical abortion were selected over a period of 14 months from January 2007 to March 2008. They were given 100 mg Mifepristone orally on Day-1, followed by 800 μg Misoprostol orally 24 hours later on Day-2, keeping the patient in the ward for at least 6 hours.

Abortion interval, success rate, post-abortion bleeding and side-effects were noted. Success was defined as complete uterine evacuation without the need for surgical intervention.

**Results:**

The total success rate of this minimal dose Mifepristone regimen was 96.25%. Pain and nausea were the predominant side-effects noted. In total 72 (90%) women had completely aborted within 5 hours of taking Misoprostol. Three (3.75%) women only required suction aspiration, hence termed as failed medical abortion. The abortion interval increased with the gestation age. All three failures were of the more-than-42-day gestational age group. The overall mean abortion interval was 4.68 ± 5.32 hours.

**Conclusion:**

Mifepristone 100 mg, followed 24 hours later by Misoprostol 800 μg orally, is a safe and effective regimen for medical abortion.

## Introduction

Abortion, or the termination of pregnancy, is an important aspect of obstetric practice. Globally, around 40–60 million abortions occur annually. In developing countries unsafe abortions by untrained persons are one of the major causes of maternal mortality.

Medical abortion offers an alternative to surgical evacuation, for women with early pregnancy who wish to avoid a surgical procedure. Initially in 1970s prostaglandins alone, in high doses were tried. Mifepristone (RU-486) the anti-progestin, became available in early 1980s and later it was found that, when used in conjunction with prostaglandins, especially Misoprostol (PGE-1 analogue) it completes the abortion procedure without instrumentation. Since then researchers have been attempting to determine the effective dosage schedule of both Mifepristone and Misoprostol with maximum success rate.

Initially 600 mg Mifepristone was tried for medical abortion which was later reduced to 200 mg. In this study we have endeavoured to determine the efficacy of 100 mg Mifepristone.

### Setting

#### Key focus

The key focus is to decrease the dose of Mifepristone which reduces the cost of treatment and its side-effects. The regimen is modified by decreasing the time interval to 24 hours (instead of the recommended 48 hours) of Misoprostol after Mifepristone to make it short and patient-friendly.

We have also strived to minimise hospital visits and hospital stays to make the regimen even more cost-effective.

#### Background

The initial French protocol was that of 600 mg Mifepristone on Day-1, followed by Misoprostol 600–800 µg 48 hours later on Day-3. Nowadays WHO (World Health Organisation) recommends a reduced Mifepristone dose of 200 mg on Day-1. This is followed by Misoprostol 800 μg vaginally 48 hours later on Day-3, with 98.5% success in less than 49 days and 96.5% for 50–63 days gestational age cases.

#### Objectives

The objective of the study was to determine the efficacy of low dose (100 mg) Mifepristone for medical termination of early pregnancy with Misoprostol 800 μg (PO), at 24 hours interval only.

#### Contribution to field

The study results will provide the new low dose regimen for the purpose of medical abortion in a short time span.

#### Research significance

In this study, a trial is carried out to reduce the dose of Mifepristone for early first trimester abortions. At the same time, it is attempted to decrease the time interval between the two drugs, Mifepristone and Misoprostol, so that regime can be safer, patient-friendly, and cost-effective.

## Ethical consideration

Approval was obtained College Ethical Committee before the start of the research project work. All the patients enrolled for the study was first informed about the new drug protocol and written informed consent was obtained prior to medication. Women who refused the new minimal dose regimen were kept on WHO recommended 200 mg Mifepristone (PO), which was followed by Misoprostol, vaginally, after 48 hours.

### Potential benefits and hazards

#### Benefits

A low dose regimen will decrease the side-effects of the drugs and will reduce the total cost of the treatment. If found to be successful, patients can be advised to take the medicines at home and report in case of problem, and consequently reduce hospital visits.

#### Hazards

The dose of the drug Mifepristone is being reduced and as such there is theoretical chance of a high failure rate or risks of incomplete abortions.

### Recruitment procedures

Patients who attended the family planning and gynaecology OPD (Outpatient Department) at the Rural Institute of Medical Sciences And Research, Saifai, to request the termination of their pregnancy up to 56 days of gestational age from their last menstrual period, from January 2007 to March 2008, were selected for the study.

## Method

### Materials

The study included 82 women of reproductive age, opting medical abortion as a mode for termination of early pregnancy.

### Settings

#### Prospective study

A prospective clinical trial with lower drug dosage is advisable instead of present recommendations for medical abortion.

### Procedure

At the first visit of the patient, a detailed history was taken and a general, systemic and obstetric examination was done. The duration of the pregnancy was calculated from the last menstrual period (LMP) and correlated with a pelvic examination. An ultrasound was carried out to confirm the gestation age (inclusion criteria of less than 56 days) and to rule out an ectopic pregnancy. Routine investigations such as blood grouping, a haemogram, a platelet count, bleeding time and clotting time were carried out. An informed consent was obtained after counselling in regard to the drug dosage schedule, hospital visits, the advantages and possible side effects and warning signs so that they could call and reach the hospital in time, in case of an emergency. Patients with coagulation disorders, medical diseases such as diabetes, asthma, jaundice, heart disease, severe anaemia and those with a gestation age of more than 56 days (8 weeks), were excluded from the study.

A patient was given a tablet of Mifepristone (RU-486) 100 mg on Day-1 after completing the work-up and she was asked to return the next day (Day-2), 24 hours later, when four tablets of Misoprostol (200 µg each) were administered orally with a tablet of Metoclopramide. The patient was kept in hospital under observation for at least 6 hours. Vital signs, the time of onset of pain, as well as bleeding and expulsion of products of conception were recorded. Patients who did not abort with in 24 hours were given a repeat dose of Misoprostol 400 µg orally. A digital examination was carried out to monitor the amount of bleeding and to check the completeness of expulsion before the patient was sent home with the advice return for a follow up after 14 days (Day-15), or earlier in case of severe pain or excessive bleeding. Patients that had no bleeding even after the second Misoprostol dose were termed ‘Failed medical abortion’ and suction evacuation was performed. Mean, standard deviation and CI (Confidence Interval) were used as statistical methods.

## Results

Two out of the total 82 patients recruited for the study, were lost as they absconded in the post-Misoprostol-observation period and did not even return for a follow up. The majority of the patients were 20–30 year old married women with a lower-middle and middle socio-economic status.

Nine patients were primigravidas and, of those, four (5.00%) were unmarried (Table 1).

**TABLE 1:** Patient characteristics (*n* = 82).

S. No.	Parameters	Characteristics	*n*	%	Mean ± s.d.
1	**Age**	Years	-	-	26.4 ± 6.45
2	**Gravidity**	Gravida 1	9	-	-
		Gravida 2–3	42	-	-
		Gravida 4 or more	31	-	-
		Gravida	-	-	3.03 ± 1.22
3	**Marital status**	Married	78	95	-
		Unmarried	4	5	-
4	**Gestational age**	< 35 (days), 5 (weeks)	8	-	-
		≥ 35–41 (days), 5–6 (weeks)	36	-	-
		≥ 42–48 (days), 6–7 (weeks)	20	-	-
		≥ 49–56 (days), 7–8 (weeks)	18	-	-

*Source*: Authors’ original data S, serial (number); *n*, given as means of number; s.d., standard deviation.

About 58 (72.5%) patients requested termination of their pregnancy as they already had to manage a previous baby of less than two year old. The remaining 13 (16.25%) patients were pregnant because of the couple's irregular use of contraceptive pills and condoms. Most of the patients were of the 35–42 days gestational age group.

Completely successful medical abortion took place in 77 (96.25%) patients out of which three patients (3.75%) required a repeat dose of 400 µg Misoprostol after 24 hours. Seventy-two (90%) women had a complete abortion without any surgical intervention within 5 hours of taking Misoprostol (Table 2). Three (3.75%) women only required suction evacuation, hence termed as failed medical abortion. Nine patients had a small amount of bleeding after taking Mifepristone, but no one had complete expulsion before taking the Misoprostol.

**TABLE 2:** Medical abortion outcome (*n* = 80).

Abortion Interval after Misoprostol (hours)	Additional action required	*n*	%	Cumulative (%)
< 3	-	18	22.50	22.50
≥ 3–5	-	54	67.50	90.00
≥ 5–8	-	2	2.50	92.50
≥ 24 hours	Repeat dose of Misoprostol 400 µg.	3	3.75	96.25
Failed cases	Not aborted; required suction evacuation	3	3.75	-

*Source*: Author's original data *n*, given as means of number.

The mean abortion interval increased with an increase in the gestational age at the time of seeking the abortion (Table 3). The overall mean abortion interval was found to be 4.68 ± 5.32 hours. The mean duration of vaginal bleeding afterwards was 6.01 ± 3.90 days (CI; 3–18 days). A positive correlation was found between the duration of bleeding and the preceding days of amenorrhea.

**TABLE 3:** Gestation age versus abortion outcome.

Gestation age (days)	*n*		Mean ± s.d. (hours)	
	
	Recruited	Complete follow-up	Abortion interval after Misoprostol	Post-abortion bleeding days
< 35	8	8	2.72 ± 0.10	6.37 ± 2.68
≥ 35–41	36	36	3.23 ± 0.49	5.94 ± 1.82
≥ 42–48	20	19	5.37 ± 5.80 1 patient required a repeat dose	9.73 ± 3.75
≥ 49–56	18	17	7.77 ± 8.77 2 patients required a repeat dose	7.64 ± 4.27

**Total**	**82**	**80**	**4.68 ± 5.32**	**6.01 ± 3.90**

*Source*: Authors’ ariginal data

*n*, given as means of number; s.d., standard deviation.

Nausea and abdominal pain were the predominant side-effects noted during the study. Five patients (6.25%) had diarrhoea and two complained of fever, the fever being more than 37.8 °C (100 °F) later on. Most of the patients stopped bleeding 7–10 days after the abortion. No patient required a blood transfusion.

## Discussion

The anti-progestin, Mifepristone (RU-486), causes abortion by competitively blocking progesterone receptors and effect is maximized by adding prostaglandin, especially Misoprostol which is inexpensive, stable at room temperature, and can be administered by different routes and thus provides better patient compliance.

In 1999, the International Federation of Obstetrics and Gynaecology (FIGO) stated:

after appropriate counselling a woman has the right to have access to medical or surgical induced abortion, and … Healthcare services have an obligation to provide such services as safely as possible.

(RCOG clinical guidelines 2004)^1^

More recently, WHO has added Mifepristone and Misoprostol to its list of essential medicines for developing countries, as WHO believes that these medicines ‘satisfy the priority health care needs of the population’.^2^

A total success rate of 96.25% was recorded with 100 mg Mifepristone in our study, which is quite similar to the success rate documented by other researchers (Table 4). Creinin et al.^3^ had reported a success rate of 85% in medical abortions with 100 mg Mifepristone plus 400 µg oral Misoprostol, and 95% success with 800 µg vaginal Misoprostol up to 49 days gestational age. Jerbi et al.^4^ has reported 94.4% success with 100 mg Mifepristone and 400 µg vaginal Misoprostol.

**TABLE 4:** Comparative success rates of medical abortion by different regimens.

S. No.	Author and year	Patients (*n*)	Gestation (days)	Mifepristone Dosage (mg)	Interval time between two drugs (hours)	Misoprostol dosage		Success Rate (%)	95% CI

						Oral	Vaginal				
1.	Peyron, R^7^ 1993	505	49	600	48	400	-	96.90	94.10–97.70
2	Takkar, D^9^ 1999	51	56	200	48	600	-	88.63	-
3	Ashok, PW^10^ 1998	2000	63	200	36–48	-	800	97.50	-
4.	Schaff, EA^6^ 2000	2255	56	200 200 200	24 48 72	- - -	800 800 800	98.00 98.00 96.00	97.00–99.00 97.00–99.00 95.00–97.00
5.	Creinin, MD^3^ 2001	40 40	49 49	100 100	48 48	400 -	- 800	85.00 95.00	71.00–94.00 85.00–99.00
6.	Lin M^8^ 2005	356	49	200	48	600 SL	-	98.30	-
7.	Jerbi M^4^ 2005	762	56	100	48	400	-	94.40	-
8.	Present study 2007–2008	82	56	100	24	800 Extra 400	-	92.50 96.25	87.80–97.10

Note: Please see the full reference list of the article, Seth S, Nagrath A, Goel N, Low dose Mifepristone (100 mg) for medical termination of pregnancy. Afr J Prm Health Care Fam Med. 2011;3(1), Art. #254, 5 pages. doi:10.4102/phcfm.v3i1.254, for more information. *n*, given as means of number; S, serial (number); CI, confidence interval.

Efficacy decreases and the abortion interval increases with an increasing gestation age, which may be somewhat improved if we administer Misoprostol by vaginal route, because of the greater local tissue bioavailability and higher uterine activity (Montevideo units) by vaginal route. A high dose of Misoprostol orally, however, produces peak plasma levels earlier, and thus can help theoretically in early evacuation (Misoprostol kinetics by Zeimen).^5^

The conventional timing of Misoprostol administration after Mifepristone for medical abortion is 2 days, but more flexible intervals which implies a more convenient regimen, was tried. In this study we gave Misoprostol 24 hours after Mifepristone which consequently decreased the duration of the total abortion process. Schaff et al.^6^ had documented 98% complete abortion rate with Misoprostol (vaginally) after 1 day, 98% after 2 days and 96% after 3 days of Mifepristone. This suggests that Misoprotol need not to be administered strictly after 48 hours.

One drawback of this method of pregnancy termination is the inconvenience of the required stay of 6 hours in the clinic after the administration of Misoprostol. Most adverse events, however, including those rated as severe, occur during this period which can be dealt with easily and some women may prefer to be in the clinic during these events.

It was observed that patients with less than a 42-day gestational age had a mean bleeding duration after abortion of approximately 6 days, whilst those with more than a 42- day gestational age had a mean bleeding duration of 7–9 days. Peyron, Aubeny, Targosz, et al.^7^ had reported a definite positive correlation between the duration of amenorrhea preceding the administration of Mifepristone and the duration of bleeding after abortion. It follows that the earliest possible intervention is recommended after the decision to have an abortion has been made. This regimen can be tried for a home-based medical abortion prescription without side-effects after a complete workup, but further and larger studies are required.

## Conclusion

Low dose Mifepristone (100 mg) combined with oral Misoprostol is an effective alternative 'to surgical intervention’. Proper counselling about the procedure to be followed, as well as information about possible high-risk symptoms must be communicated to the patient beforehand. An observation period of 4–6 hours after the administration of Misoprostol is a better option as maximum pain and bleeding occurs during that time. Furthermore the doctor remains sure of expulsion, and a digital per vaginal examination confirms it as well.
